# Perceived mental illness stigma among family and friends of young people with depression and its role in help-seeking: a qualitative inquiry

**DOI:** 10.1186/s12888-022-03754-0

**Published:** 2022-02-11

**Authors:** Ellaisha Samari, Wen Lin Teh, Kumarasan Roystonn, Fiona Devi, Laxman Cetty, Shazana Shahwan, Mythily Subramaniam

**Affiliations:** Research Division, Institute of Mental Health, Buangkok Green Medical Park, 10 Buangkok View, 539747 Singapore, Singapore

**Keywords:** Depressive disorders, Help-seeking, Stigma, Young people, Qualitative

## Abstract

**Background:**

Depressive disorders are a serious public health concern. Left untreated, further clinical distress and impairment in important life domains may arise. Yet, the treatment gap remains large. Prior research has shown that individuals with depressive disorders prefer seeking help from informal sources such as family and friends ahead of formal sources. However, this preference has its disadvantages such as experiencing actual, perceived and internalized stigmatizing responses from them which may delay or deter help-seeking. This paper aimed to determine the role of perceived stigma among family and friends in an individual’s help-seeking behavior.

**Methods:**

Data were collected using semi-structured interviews with patients with depressive disorders from a tertiary psychiatric hospital in Singapore to capture individuals’ self-reported experience with depression and stigmatization among family and friends. Interviews were audio recorded and transcribed verbatim. Data of 33 young adults (mean age = 26 years, SD =4.6; 18 female, 15 male) were analyzed using thematic analysis.

**Results:**

In all, four broad themes were developed: (1) absence of support, (2) provision of unhelpful support, (3) preference for non-disclosure, and (4) opposition towards formal help-seeking. Lack of awareness of depression and perpetuation of stigma manifests as barriers towards help-seeking in the form of absence of support and provision of unhelpful support which subsequently leads to a preference for non-disclosure, as well as opposition by family and friends towards formal help-seeking.

**Conclusions:**

Data from this study can contribute to the development of public health programs aimed at improving awareness and support from family and friends and facilitating earlier help-seeking among young people with depressive disorders.

**Supplementary Information:**

The online version contains supplementary material available at 10.1186/s12888-022-03754-0.

## Introduction

Depressive disorders remain a serious public health concern. Based on a report by the World Health Organization (WHO) in 2017, an estimated 4.4.% of the world’s population have depression, of which approximately 27% (86 million) reside in Southeast Asia [1]. Symptoms of Major Depressive Disorder (MDD) can result in significant clinical distress and impairment in important life domains such as employment and interpersonal relationships [2]. Left untreated, further problematic consequences may arise. A systematic review and meta-analysis conducted by Ghio et al. [3] summarized that a longer duration of untreated illness is a strong predictor of poor response to anti-depressant treatment, lower rate of remission, higher risk of chronicity, as well as higher number of recurrences. This renders timely and appropriate help-seeking of paramount importance in an individual’s journey to recovery. Yet, treatment gaps remain large, with common barriers to help-seeking being cost of treatment, stigmatization, and lack of perceived need for treatment [4–7].

Rickwood et al. [8] had defined help-seeking for mental health problems as “an adaptive coping process that is the attempt to obtain external assistance to deal with mental health concerns”. This includes seeking assistance from both formal (e.g., health professionals or services) and informal (e.g., friends and family) sources. Prior research has shown that individuals with depression prefer seeking help from informal sources such as from family and friends ahead of support from formal sources [9–11]. This preferred source of support is unsurprising as there are some perceived advantages from consulting family and friends such as receiving emotional, informational, and instrumental support [12, 13]. Moreover, perceived positive support from informal sources has been associated with improved recovery among individuals with a depressive disorder [14]. However, there are some concerns that seeking help from informal sources can lead to unhelpful or harmful consequences, such as experiencing actual, perceived and internalized stigmatizing responses [12]. As interaction with family and friends are likely to be more frequent, they are at a crucial position to provide emotional and informational support which facilitates formal help-seeking, which is, help from health professionals [13]. In contrast, stigmatizing responses from family and friends may delay or deter individuals from seeking help from both informal and formal sources.

Stigma is a distinctive feature associated with mental illness [15]. According to Corrigan et al. [16], stigma consists of three related concepts: stereotypes, prejudice, and discrimination. Stereotypes, which tend to be culturally determined, are oversimplified ideas including preconceptions about traits or abilities of people belonging to a specific group. It emphasizes the differences between people from this group and another group; consequently, drawing people to focus on information that aligns with the stereotypes and ignoring those which do not. Prejudice on the other hand is a negative attitude based on stereotypes towards people belonging to a specific group. Stereotypes and prejudice can lead to discrimination, which is the unjust behavior towards people belonging to a specific group. In the context of mental illness, stigma of mental illness can create social distance or rejection in the form of decrease in opportunity for employment (discrimination), resulting from negative labels (stereotype) placed on people with mental illness (e.g., unstable, dangerous, and unpredictable), and fear of them (prejudice). Consequently, such stigma can discourage individuals from seeking treatment due to the anticipation of being labelled with a mental illness and being discriminated against [17].

Stigma towards mental illness is pervasive and cultural contexts may shape its form [18]. In Asia, mental illness is often associated with stigma, shame and “loss of face” (losing the respect of others) for the individual and their family members [19, 20], which could be partly due to the Asian values of collectivism [21]. In Singapore, research has identified similar associations such as perceiving that people with mental illness are unpredictable and that the illness is a sign of personal weakness [22]. Furthermore, results from a study conducted among 940 youths in Singapore found that 46.2% said they would be embarrassed if they were diagnosed with a mental illness, 22.7% said they do not want others to know if they had a mentally ill relative, and 35.1% said their friends would see them as weak if they had a mental illness [23]. These collectively suggest that mental illness is seen as a mark of shame among youths in Singapore and it is something that peers would stigmatize.

Singapore is a multi-ethnic country in Southeast Asia with a population of approximately 5.7 million in 2019, comprising mainly Chinese (74.3%), Malays (13.5%), Indians (9%), and those belonging to other ethnic groups (3.2%) [24]. Based on a national survey of mental health disorders in 2016, the lifetime prevalence of MDD was 6.3% among the general population aged between 18 and 65 years and 9.2% for those aged between 18 and 34 years [25]. Despite depression being a prevalent condition and identified by youth in Singapore as one of the top issues they faced [26], a large treatment gap remains apparent [6, 25, 27]. Furthermore, the Mind Matters study – a population-based cross-sectional study conducted among Singapore residents – found that approximately half of the participants endorsed “talking to family or friends” as the most recommended source of help for depressive disorders [10].

Connectedness and group cohesion are important values in a collectivist culture [28] as are deference to authority figures and maintenance of interpersonal harmony with the family [29]; therefore, it is likely that people belonging to a collectivist culture would involve their family members in their decision making and adopt choices made by trusted others. This is echoed in prior research findings where there was a preference for family-centered decision making in mental health care among East Asians [30].

Prior research has shown that family members of those with mental illness experience associated stigma and its negative consequences. In Yin et al.’s [31] qualitative systematic review of experience of stigma among family members with severe mental illness, the authors highlighted that family members experienced social exclusion, isolation and received nasty comments that devalue and ridicule them due to their mentally ill family members. Importantly, the authors noted that some family members tried to cope with the stigma by concealing mental illness from others to avoid discrimination, or by reducing contact with others to avoid being confronted with stigmatizing reactions. Reaction and coping styles from families can in turn have implications on the way individuals with mental illness cope with their illness.

Aside from the family's response, exposure to stigmatizing responses from peers towards mental illness too can influence the way individuals with mental illness cope with their illness. For example, some adolescents reported censoring their need for psychiatric medication due to the perception that taking medications would make them feel different from their peers, or to avoid being outcasted or humiliated for taking them. This usually stemmed from being exposed to hearing people suggest that psychiatric medications are for people who are “crazy” [32].

As it was commonly found that informal sources are the preferred source of help for depressive disorders, and commonly recommended among Singapore residents in an earlier study that examined mental health literacy [10], it is worthwhile to explore *how* stigma towards depression among informal sources in this cultural setting influence individuals’ help-seeking behavior. Such data would be useful for interventions or educational programs aimed at improving recognition and support by informal networks of individuals with depression.

Data from this paper is based on secondary analysis of a study by Teh et al. [33] who examined Singaporean young adults’ narratives of their experiences with depression, with a focus on their perceptions of the illness. The authors found that young adults typically experienced depression as a ‘reduced state of being’ and faced conflicts between the self and their social environment. The paper also brought forth important sociocultural nuances in the understanding of and experience with depression, such as their struggles to meet unrealistic societal expectations and construct meaningful goals that are aligned with familial and societal expectations as key contributors to depression. The present study thus hoped to expand on their research by delving deeper into the implications of sociocultural factors in help-seeking behaviors and experiences. Using qualitative methods, the aim of this study was to explore the role of stigma from family and friends in help-seeking behaviors among young people with depression. Our research questions include: (1) Are individuals with depression stigmatised by their family and friends? and (2) How do these experiences delay or deter help-seeking?

## Methods

### Study Design

The present study applied a qualitative methodology. Following an interpretative approach [34], semi-structured interviews were used to obtain narratives of young people’s experiences with stigma from their family and friends and its role in help-seeking. The study was carried out in accordance with the latest version of the Declaration of Helsinki and ethics approval was obtained from the National Healthcare Group Domain Specific Review Board (NHG-DSRB) and the Institute of Mental Health, Institutional Research Review Committee (IRRC). All participants provided written informed consent prior to their participation. Reporting of this study was guided by the consolidated criteria for reporting qualitative studies (COREQ) [35] (see Additional file 1: Appendix A).

#### Participants and recruitment procedure

Participants were recruited from the Institute of Mental Health (IMH), a tertiary psychiatric hospital in Singapore between February 2018 and January 2019. Inclusion criteria for the study were: being a Singapore citizen or permanent resident, aged between 18 and 35 years, receiving treatment at IMH outpatient clinics, having a diagnosis of depressive disorder, and willing and able to give written consent. Participants were excluded if they had a diagnosis of substance-induced depressive disorder, depression with psychotic symptoms, bipolar disorder, depressive disorder due to a general medical condition, or were women with post-natal depression. Participants were recruited via convenience sampling and purposive sampling to obtain adequate representation by ethnic groups. No relationship was established between participants and study team members prior to study commencement. Participants were either referred by their clinicians during their outpatient visit or inpatient stay; in the latter instance, they were recruited during outpatient follow-up or approached at the outpatient clinic by a study team member with information flyers detailing the aim, procedures, criteria and potential risks and benefits of the study. Of the 52 individuals approached, 14 refused to participate. Some did not mention a reason for refusal while others said they were disinterested, busy, or uncomfortable with being interviewed. Four participants were withdrawn as they did not fulfil the eligibility criteria. 34 participants (19 females, 15 males) completed the interviews. One transcript was excluded from analysis as it differed substantially from the rest of the transcripts where discussions were primarily associated with another medical condition.

#### Data collection

 Face-to-face semi structured interviews were conducted with participants by study team members [ES, WLT, KR, FD, LC, and SS] who are researchers with degrees in psychology (i.e., BA/BSc in Psychology, Masters in Clinical Psychology), trained in qualitative research and had prior experience in conducting qualitative interviews. Study team members consisted of both males and females. As the communication styles of individuals in Asian cultures differ substantially from Western cultures, one-to-one interviews were preferred over focus group discussions [36]. A semi-structured interview format was also chosen to guide the direction of the interview yet remaining flexible to allow follow-up of noteworthy areas of accounts which may surface during the interview. Semi-structured interview questions from the larger study by Teh et al. [33] included: What do you personally believe led you to being depressed? How has depression affected you? How have you coped with depression? What does your family or culture think of depression? Does the way your family or culture think of depression affect the way you think of depression? Follow-up prompts (Can you tell me more about it? Could you give me an example of this?) were also used.

All interviews were conducted within the IMH facility except for one interview which was conducted outside IMH for the convenience of the participant. Only the participants and researchers were present during the interviews. To ensure sensitivity to participants’ ethnocultural perspectives, most participants were matched with interviewers of similar ethnicity. Interviews ceased once data saturation was reached, where no new information was observed and collected. Interviews were audio-recorded, transcribed verbatim and checked for accuracy by study team members. Each interview lasted between approximately 33 and 120 min (average 63 min).

### Data analysis

Transcripts were pseudonymized via assignment of an alphabet (C, M, I) based on ethnicity followed by a number in sequence of recruitment for that ethnic group (e.g., C01, M01, I01). Transcripts were uploaded onto Nvivo software, a qualitative data analysis software, for data management and analysis [37].

Analysis was undertaken by five study team members [ES, WLT, KR, FD, and LC]. In analyzing the data, a combination of inductive and deductive approaches was used where data collection and analysis happened simultaneously and iteratively [38, 39]. For the inductive approach, the study team members independently read each transcript and collaboratively extracted and coded meaningful data units using the open coding method [40]. Initial codes were generated into higher order concepts and themes based on their common properties and a codebook was developed. The study team discussed and reviewed key emergent themes extensively while constantly comparing new data with previously collected data as part of the deductive approach where pre-existing codes were used as a guide and template for the clustering of newly collected data. The codebook was regularly refined until data saturation was achieved where no additional data were found to develop new themes. Using Nvivo, five coders [ES, WLT, KR, FD, and LC] coded the same transcript and their coding was compared using the coding comparison function which yielded an average kappa score of 0.73, indicating high inter-rater agreement among the five coders.

The next step was performed by two study team members [ES and WLT] which consisted of examining one of the key themes identified in the data. This theme related to participants’ experiences with their family and friends’ reactions towards their depression. ES and WLT further classified data from this theme into distinct but interrelated themes using the thematic analysis approach as informed by Braun et al. [41] to identify, analyze, and report patterns within the data set. This approach was chosen as it is particularly suited to interpreting the conceptualization of a phenomenon by a specific group [42]. Using NVivo as a data management tool, ES and WLT generated initial codes and sub-codes in a systematic fashion (see Additional file 2: Appendix B), which were then grouped into potential themes. Transcripts were consistently checked to ensure that quotes were congruent with the themes.

## Results

Data for the present study were gathered from interviews with 33 participants (18 female, 15 male) between the ages of 20 and 35 years (mean = 26; SD = 4.6). All participants were outpatients who had a diagnosis of depressive disorder according to the Diagnostic and Statistical Manual, 4th Edition (DSM-IV); two of whom had comorbid depression and anxiety. Duration of depressive disorders varied from four months to 16 years, with a median of two years. The participants came from a variety of educational and employment backgrounds: 26 participants graduated from vocational school, junior college, or higher, and seven with secondary school education or lower; 7 were students, 18 had full-time or part-time jobs, and five participants were unemployed at the time of the interview. The overview of participants’ characteristics can be seen in Table 1.

**Table 1:** Participant characteristics, diagnosis, and other sociodemographic information (n = 33)

S/N	Age	Sex	Ethnicity	Primary diagnosis	Years with depressive disorder	Religion
C01	34	Female	Chinese	MDD	8	Buddhism
C02	20	Female	Chinese	Anxiety depression	<1	Christianity
C04	30	Male	Chinese	MDD	2	Buddhism
C05	34	Female	Chinese	Mixed anxiety and depression	7	Christianity
C06	26	Male	Chinese	MDD	8	Christianity
C07	24	Female	Chinese	MDD	<1	Taoism
C08	31	Female	Chinese	Dysthymia and MDD	3.5	Christianity
C09	35	Female	Chinese	Depression	<1	Buddhism
C10	22	Female	Chinese	MDD	4	Christianity
C11	22	Male	Chinese	MDD	2	Buddhism
C12	22	Female	Chinese	Depression	2^	Buddhism
C13	20	Male	Chinese	MDD	3	Free-thinker
L01	34	Female	Indian	MDD	16	Hinduism
L02	27	Male	Indian	MDD	7^	Buddhism
L03	27	Female	Indian	Depressive disorder	4	Hinduism
L05	21	Male	Indian	MDD	1	Free-thinker
L06	26	Male	Indian	Depression	2	Christianity
L08	22	Female	Indian	Dysthymia	<1	Free-thinker
L09	26	Female	Indian	MDD	8	Islam
L10	25	Male	Indian	Dysthymia	2	Others
L11	23	Male	Indian	Depression	7	Christianity
L12	22	Female	Other	MDD	6	Free-thinker
M01	26	Male	Malay	Reactive depression	3	Islam
M02	23	Male	Malay	Depression	1	Islam
M03	27	Male	Malay	Depression	<1	Islam
M04	21	Male	Malay	MDD	1	Islam
M05	28	Male	Malay	Depression	2	Islam
M06	22	Female	Malay	Major Depression	4	Islam
M07	25	Female	Malay	Dysthymia	<1	Free-thinker
M09	35	Female	Malay	Depression	2	Others
M10	29	Female	Malay	Depression	2	Islam
M11	31	Female	Malay	MDD	3	Islam
M12	26	Female	Malay	MDD	1	Islam

Note: ^ refers to approximate number of years diagnosed with depressive disorder reported by the participant

In all, four interrelated broad themes emerged from the data: (1) absence of support, (2) provision of unhelpful support, (3) preference for non-disclosure by individuals with depression, and (4) opposition towards formal help-seeking, each encapsulating the manifestations of stigma by family and friends towards depression as barriers to help-seeking for mental health issues. The overview of themes and sub-themes can be seen in Table 2. To ensure that standard usage of English is maintained, minimally corrected verbatim of quotes are shown.

**Table 2:** Overview of major themes and sub-themes

Major themes	Sub-themes
Absence of support	1.1. Ignoring cries for help 1.2. Brushing off symptoms with insensitive remarks
Provision of unhelpful support	2.1. Inappropriate comparisons with other experiences 2.2. Providing unsolicited or unhelpful advice 2.3. Attributing depression to one’s character flaws
Preference for non-disclosure	
Opposition towards formal help-seeking	4.1. Inability to accept the illness 4.2. Distrust of psychiatric practice

### Absence of support

A major finding from this research is the absence of support by family and friends. This absence is likely driven by their misconceptions of the illness, such as believing that depression is not a serious or real medical condition, or shame associated with the illness.

#### Ignoring cries for help

A few participants had mentioned the inability of family and friends to recognize the severity of their depressive symptoms and had instead ignored or trivialized their experiences.

*“they’re not doing anything to help because to them it’s like, ah depression, my friend also got depression, my father also got depression, nothing like… to them it’s nothing serious” (M01/26/M/Malay)*.

On the other hand, some were more explicit in sharing their family’s denial of the illness as the cause of their inaction towards cries for help.

“*My brother told my mom that he’s not okay but to my parents it’s just like your life is not so…so messed up for you to be depressed. Like she said like Princess Diana she controls the whole country, she has the right to be depressed. You are just a normal boy, you cannot have depression. Like they can’t accept their children having depression. But the thing is – yeah, I understand – but the thing is we voiced out for help, we are crying for help already so you need to do something about it and not just because you don’t want other people to know that my children got depression.” (C07/24/F/Chinese)*.

*“My father is like kind of clueless about it. He’s like in denial? “Oh, she’s alright, she’s alright.” Like I’m sitting down there, crying “oh, you’re alright, you’re alright” like in denial that kind of thing.” (M10/29/F/Malay)*.

#### Brushing off symptoms with insensitive remarks

 Some participants expressed being brushed off with dismissive remarks that minimized and even normalized their experiences when they sought help from family and friends.

*“Even if I told them, they wouldn’t take it seriously. Like after I tried to kill myself, I went to them and then they simply brushed it aside, just as a reaction to stress.” (L05/21/M/Indian)*.

Akin to the example above, some of the dismissive remarks experienced by participants suggests the lack of belief in depression as a real and chronic medical condition among family and friends. In fact, many of the participants’ narrations indicate that depression was instead seen as merely an experience of regular emotions:

*“they don’t see it as an illness; they see it as emotions you see. So that’s why they were like oh you feeling like this (it’s) you giving up, it’s just you…” (M03/27/M/Malay)*.

Or an individual’s way of inflating their present situation for their own personal gains such as to avoid responsibilities:

*“…it's just frowned upon on like people who try to chao keng (malinger), like they go and fake MC (medical certificate) for something or what. So there’s always this belief that people with mental illnesses are just chao keng-ing (malingering) ah, they are just faking it”(C11/22/M/Chinese)*.

### Provision of unhelpful support

Another major finding includes the provision of unhelpful support by family and friends – a manifestation of ignorance and negative attitudes towards depression – and the resultant feelings of the participants.

#### Inappropriate comparisons with other experiences

 It was noticeable in their recounts that participants yearned for empathy and a ‘listening ear’ from family and friends. However, family and friends oftentimes tried to relate participants’ experiences with their own or experiences of others. Although these responses were in some instances well-intended, participants felt that their situations were different and were unable to gain insights from those comparisons, and even found them unhelpful.

*“I will tell them I’m having trouble sleeping or I’m having trouble concentrating and they’ll be like “yes, yeah, me too…I’m also very stressed about exams.” But then after exams they are all like ‘La Di Da’ happy but I’m still feeling the same,”(L09/26/F/Indian)*.

On a related note, another participant described her friend’s attempt to make sense of her situation but in doing so, invalidated her experience and emotions by comparing it with another situation which was perceived to be worse:

*“I kind of find it annoying when people like tell me, like other people have it worse. Showed me pictures of hungry children, “See they having it worse why you feeling like that?” You know that kind of thing? Every time I feel sad, people will also, “You see, got children hungry. You are here got food, got a roof over your head. Why you sad?” You know? You know all this sort of thing? So it’s like, a comparison.” (M10/29/F/Malay)*.

Comparisons with situations that were deemed to be worse were also seen in other narrations, usually with previous generations’ experiences of having to deal with seemingly tougher situations yet not experiencing depression; as though alluding to the lack of strength of one’s character as an explanation for depression.

* “In the past they (parents) did not have money, or roof over their heads, but they are able to survive, and how is it that youngsters nowadays with food and shelter over the head, got mental problems?” (C10/22/F/Chinese)*.

#### Providing unsolicited or unhelpful advice

Echoed among participants was an inability by family and friends to react to their illness in a manner which participants found useful. Instead, they were given unsolicited advice over being listened to and empathized with, which was the response most participants had sought for.

*“I understand from another point where you know you give a lot of advices and stuff but I feel like we don’t really need them. We don’t really need advice we just need you to listen and understand how we feel…” (C10/24/F/Chinese)*.

Among the unsolicited or unhelpful remarks which showed an inability to fully understand the nature and etiology of depression, were participants being asked to negate their depressive symptoms (e.g., “don’t be stressed” (C05/34/F/Chinese), “just don’t feel sad” (L08/22/F/Indian)), to not dwell on them (e.g., “it’s nothing if you don’t think about it” (M12/26/F/Malay)) or to “snap out of it” (C10/22/F/Chinese). Participants also found it particularly unhelpful when they were encouraged to simply think positively (e.g., “be more positive, be stronger” (C11/22/M/Chinese), “there’s a light, just be patient” (M09/35/F/Malay)).

It was also common among family and friends of Malay participants to advise them to pray and strengthen their faith to overcome depressive symptoms. These advice tend to stem from the belief that depression was due to weak faith, supernatural causes, or that divine intervention can alleviate depressive symptoms. Although well-intentioned, participants felt that they needed more than just prayers to help their condition.

*“So therefore she (mother) believes like the more you pray, the more you’re praying the sins away and like you make better decision and stuff like that.” (M07/25/F/Malay)*.

*“The first question I usually get, “Why? What makes you depressed?” or like “Ingat Allah (Remember God)”, basically they always link it back to religion or there’s something wrong with you like, like go for, like they think got Satan or something like that if that makes sense.”(M06/22/F/Malay)*.

#### Attributing depression to one’s character flaws

A few participants spoke of attempts from family and friends to rationalize their experiences of depressive symptoms by placing blame on their character. Participants were typically assumed to be “lazy”, “uninspired”, or “weak”. In particular, a participant was scolded for showing depressive symptoms:

*“They (family) were not like accepting of uh seeing me at my worst and then their way of dealing with it was to like scold me, maybe to them they think they can, they can… like wake me out of it with scolding but sure if it’s just… if it’s a lazy person maybe you can. I couldn’t help but think that they just keep choosing to think that actually I am lazy, I’m being lazy…” (C08/31/F/Chinese)*.

### Preference for non-disclosure

Family and friends’ stigmatizing beliefs and unsupportive or unhelpful responses towards their depression in general or remarks on how individuals can overcome their condition had discouraged some participants from seeking help. In particular, some found these reactions to lack empathy, understanding, or to be insensitive, which resulted in the experience of negative emotions such as feeling disheartened, frustrated, exhausted, and annoyed.

*“And I’ll feel very tired to explain again. I’ll just mm… like that. Then like cause sometimes giving the same response over, and over again, it tires you out.” (C12/F/22/Chinese)*.

these reactions had also resulted in some individuals preferring to mask their illness, not talk about it, and rely on themselves instead, which led to the worsening of depressive symptoms for some.

*“…they’re just telling me “no you just lazy, you just sad, you just erm you’re not finding… you’re not inspired you know, bla bla bla bla” and then the more I feel defeated, the more depressed I feel, so the more I hide it.”(M09/35/F/Malay)*.

*“The reaction to mental illness is there’s always a problem with the person, that’s why they caught it. So… I’m not as open about this. I don’t dare to tell people because I’m afraid of them knowing… I feel like I explain to them they don’t understand. They really don’t understand then what’s the point? So you’ll feel very alone in this whole illness” (C05/34/F/Chinese)*.

In addition, two participants specifically mentioned the role of stigma within their cultures in not dealing with their illness

*“Culture has influenced me in a way where I just don’t want to deal with it. Like knowing that it’s a negative thing, the more I’m like “Nah, I don’t want to deal with this right now.”(L08/22/F/Indian)*.

Or being more reluctant to open up to someone from the same ethnic group. This was due to having encountered culturally specific stigmatizing responses; therefore, finding it easier to confide in someone from a different ethnic group.

“*I’m less reluctant to open up… Like it’s easier to open up to a Chinese friend than to open up to a Malay, like no offense but like people who are really good in agama (religion) because then they will like, ya ya your faith is not strong enough, those kind.”(M06/22/F/Malay)*.

### Opposition towards formal help-seeking

Some participants were met with opposition and reluctance from their family to seek help from formal sources (i.e., health professionals) due to varying reasons including denial of illness, being ashamed of the illness, and distrust towards psychiatric practice.

#### Inability to accept the illness

Based on participants’ narrations, some families were unconvinced of the existence of their illness or were struggling to accept the illness. This denial was mostly due to perceiving depression as an embarrassment; thus, the hesitance to seek treatment or preferring to keep the illness hidden.

*“When they found out when I was admitted here (tertiary psychiatric hospital), they were angry about it and after that they want to keep it…sort of kept it secret. They don’t want people to know.” (L08/22/F/Indian)*.

*“It’s just that my family has always been very hesitant. Not because the therapists are bad but because they are just having trouble dealing with it. Or coming to terms with what they are having to do. It’s a lot of shame, it’s a lot of denial.”(M09/35/F/Malay)*.

By extension, the shame of being tagged as having a mental illness creates discomfort to seeking professional treatment and having a mental illness record in the registry. therefore,  As such, some families had wanted the illness to be managed by the individual or within the family. 

*“they were also very reluctant to send me to a hospital to get treatment. Like they wanted me to manage it on my own with the private psychiatrist or manage it on… within the family but… so I guess that’s one of the reasons why I also didn’t want to go to A&E because it’s… my parents were also kind of hesitant to do that, maybe because they’re also afraid of the mental illness record” (C11/22/M/Chinese)*.

#### Distrust of psychiatric practice

Evidence of distrust towards healthcare professionals and western medicine by family and friends was present in some of the participants’ narrations. These include questioning the authenticity and legitimacy of clinical practice by therapists and doctors and believing that monetary gains were the motivation behind their practice. In fact, some advised against taking western medicine due to fears of worsening the symptoms while some believed in alternative treatments such as spiritual healing to treat the condition instead.

*“My father even says things like ultimately at the end of the day, the doctor need patients to earn money, so like, yah…so you cannot believe all the things that the doctor say because they just want to keep you as their patient to earn money.” (C08/31/F/Chinese)*.

*“ …again they don’t, they (family), they’re really old school, they don’t agree with medication, they think I’m wasting my money on a therapist, they think that I just need to pray and then everything will be ok.” (M05/28/M/Malay)*.

**Fig. 1:**
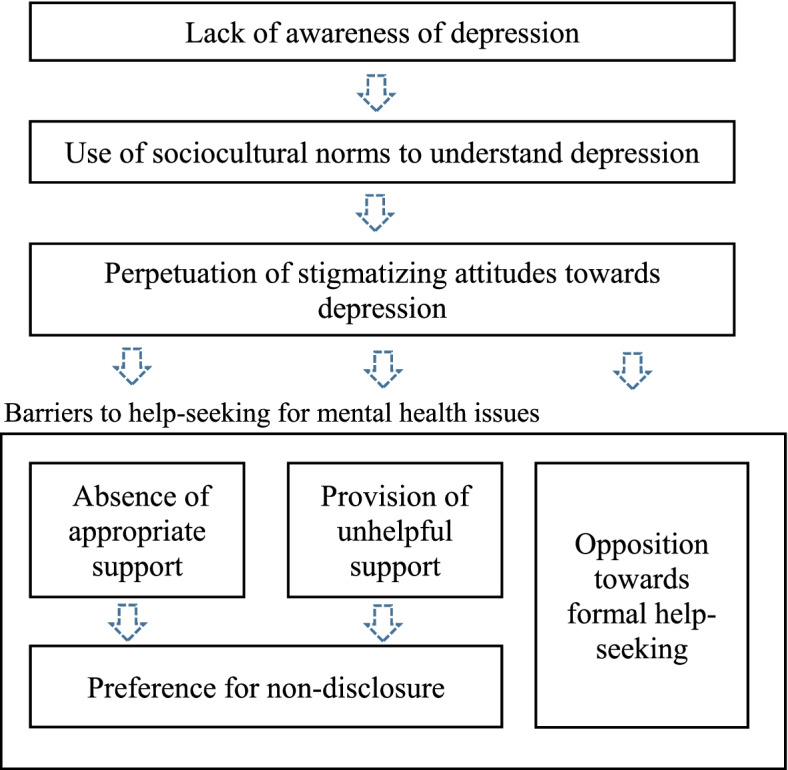
Conceptual model underlying relationship between stigma and barriers to help seeking for mental health issues

In light of the study findings, we propose a conceptual model (Fig. 1.) delineating the relationship between stigma among family and friends, and barriers towards help-seeking among individuals with depression. In short, lack of awareness of depression by family and friends results in a reliance on sociocultural norms to understand depression, which contributes to the perpetuation of stigmatizing attitudes towards depression. This in turn manifests as barriers towards help-seeking in the form of absence of support and provision of unhelpful support which subsequently leads to a preference for non-disclosure, as well as opposition by family and friends towards formal help-seeking. This model can be utilized as a guide for future research and the development of interventions or educational programs targeted at family and friends of individuals with depression.

## Discussion

Prior qualitative studies have been conducted in other countries and cultural settings on mental illness stigma among family and friends, such as the experience of associated stigma, their role in stigma development, perpetuation of stigma, and how individuals with mental illness react to the stigma (internalized stigma) [31, 32, 43]. This qualitative study conducted in Singapore examining the complex role of perceived mental illness stigma of family and friends on help-seeking adds further to the current literature in this area of research.

Findings from this study indicate the presence of considerable stigma from family and friends. While it seems unsurprising given similar findings on public stigma among community samples worldwide and in Singapore, it is worth noting that prior research has often found levels of stigma towards mental illness to be lower among those who have had contact with individuals with mental illness [22, 44]. However, results from this study reflect that stigmatization by an individual’s informal network i.e., family and friends, is still a problem and can affect the individuals’ help-seeking behaviors.

As evidenced by the narratives of participants in this study, family and friends typically respond to the illness based on their perceptions of the illness and understanding of its etiology, which tends to be based on poor depression literacy and cultural stigma of the illness (e.g., depression is just a normal emotion, depression is due to lack of faith, people with depression are lazy or weak, depression is a mark of shame, or individuals are malingering depression). This creates a problem when it delays help-seeking by affected individuals. For example, there were significant mentions of depression being attributed to supernatural elements among the Malay participants in our study, whereby a lack of faith makes a person susceptible to such disturbances; a belief that is perhaps strengthened by the practice of traditional healers who incorporate religious and cultural aspects in their treatments and whose approaches are well accepted within the community and are perceived to have encouraging treatment outcomes [45]. This observation is corroborated by a recent local qualitative study by Tan et al., [20] and a study conducted in Malaysia by Khan et al. [46]. Where supernatural elements were attributed to depression in this study, family and friends often advised participants to strengthen their faith in the religion and seek help from God to alleviate these symptoms. However, having such beliefs can be problematic when the recipient of the advice disagrees with the opinion and rejects the advice. Furthermore, advice which is perceived to lack empathy can cause them to close themselves off from others and rely on themselves instead as seen in multiple narrations in this study, which is an important barrier to help-seeking among young people [47].

While not unique to our present study [48], a rather concerning observation which this study revealed is the opposition by families and friends towards formal help-seeking. Instead, it was clear in our study that some families were keen towards suppressing the illness or keeping it hidden rather than to seek help for it. Cultural factors concerning values and norms are often cited to explain the underuse of mental health services [49, 50]. In our study, the discomfort to acknowledge depression and opposition towards use of mental health services were mainly due to shame associated with having the illness as seen with prior studies [19, 51] and not wanting others to know, which was perhaps spurred by a conservative culture [21]. In addition, the prominence of shame around mental illness here could have been due to greater emphasis on moral attribution of mental illness in Asian societies [52] and the perception that mental illnesses reflect flaws of the family [53]. Importantly, the practice of avoiding acknowledgement or discussion of subjects that are uncomfortable and shameful to talk about such as mental illness – ‘*No, this is a family thing, don’t talk about it. Shameful you know’* – then develops into a taboo subject, impeding appropriate help-seeking behavior.

Distrust of psychiatric practice is not an uncommon finding. Prior studies have shown that there are people including patients, as well as lay public, who doubt professional treatment including mental health professionals and the effectiveness of antidepressants [54–56]. Instead, preference to handle problems by themselves and unwillingness to self-disclose problems were often observed among those with mental health issues [57, 58]. In our study, participants reported that their family and friends were distrusting of professional help, specifically in terms of not believing in the legitimacy of practice of mental health professionals, and the lack of trust in western medicine to treat the conditions. This distrust can be traced back to the attributions of depression described earlier such as lack of faith and weak character thereby believing that these personal deficits should be counteracted with simply praying or strengthening one’s character, which may be unhelpful. This distrust towards psychiatric practice by families and friends can be especially tricky in instances where individuals believe that the family should decide where further help should be sought from [59, 60]. In addition, given the Asian context and their relatively young age, young people might look to their family to finalize the decisions for them.

Support from family and friends are important enablers to seeking help from a formal source. In their review of perceived advantages and disadvantages to seeking help from informal sources, Griffiths et al. [12] identified that participants received informational support from family and friends in the form of gentle encouragement and guidance towards formal sources of help. Participants also reported that family and friends assisted in evaluating their current condition and recognizing their depressive symptoms and need for professional help. Thus, informal sources of help should have adequate skills, knowledge, and depression literacy to recognize mental health issues and its need for professional help, as well as to recommend professional help when necessary. Seeking timely and appropriate professional help is essential for early detection, treatment, as well as recovery from mental illness [61, 62].

### Implications

This study revealed that stigmatization by family members is a problem and should be addressed. Based on the model (Fig. 1.) derived from the present study, interventions could be implemented to address the respective components of the model. To address lack of awareness of depression and stigmatizing sociocultural perception of the illness among family members and friends of individuals with depression, educational and anti-stigma programs that are culturally sensitive could be implemented at the population level. A population-level intervention seems appropriate given the prevalence of any mental disorders of about 1 in 7 individuals [25], where the likelihood of being a relative or/and friend of individuals with mental illness is relatively high. Furthermore, there is evidence that mental health educational programs for the public can improve mental health literacy and reduce stigma towards mental illness [63]. Key components of the program could include addressing causes of depression and perception of psychiatric practice in a culture-appropriate manner. In addition, to address the component on absence of appropriate support and provision of unhelpful support, the program could inform the public of ways to provide emotional and instrumental support in a sensitive and respectful way, highlighting the impact of their response towards the illness, and identifying various forms of support in which individuals with depression find helpful and unhelpful. Emphasis on the importance of early diagnosis and treatment leading to better outcomes should also be made.

Educational programs on providing helpful support targeting young people could also be reinforced in schools given that friends of individuals with mental illness are often sought for support yet are unable to provide appropriate help. This is especially needed considering Pang et al.’s [23] observation that a significant proportion of youths in Singapore report having little education about mental health which could explain the stigma endorsed by the sample in their study. Furthermore, there are evidence of the improvement in depression knowledge and reduction in stigma as seen in an anti-stigma intervention carried out among university students in Singapore [20].

### Limitations

Our findings should be considered in light of several limitations. Data on stigma among family and friends are limited to self-reports by young people, and therefore may not accurately capture the intricacy of the stigma and its role in help-seeking. In addition, this study did not investigate whether experienced stigma differed for individuals with different sociodemographic backgrounds. It is possible that males and females differed substantially in their patterns of response. Furthermore, we did not investigate the nature of the relationship between individuals with depression and their family and friends or their functioning and dynamics. It is also possible that differences in nature of relationship or functioning may explain varying responses and attitudes towards these individuals as well. Future research could also target caregivers and friends of individuals with depression and explore their experiences of supporting or caring for individuals with depression and their information needs.

## Conclusions

In essence, our research highlights the considerable stigma that exists among families and friends of individuals with depression. As contact with family and friends are likely to be more frequent, it places family and friends in an optimal position to facilitate earlier help seeking and improve longer term outcomes and decrease recurrences of depression. However, significant stigma towards depression which still exists among families and friends cultivates inadequate and inappropriate forms of support and delays formal help-seeking. This renders improving mental health literacy and reducing stigma among families and friends a key issue to be addressed.

## Supplementary Information

**Additional file 1.**

**Additional file 2.**

## Data Availability

Data for this study are available upon reasonable request. The data request can be sent to The Institutional Research Review Committee, Institute of Mental Health, Singapore; Email address: imhresearch@imh.com.sg.
