# Supplementary material for: Perceived mental illness stigma among family and friends of young people with depression and its role in help-seeking: a qualitative inquiry
Source: BMC Psychiatry. 2022 Feb 11;22:107. doi: 10.1186/s12888-022-03754-0 (PMC8832742; doi:10.1186/s12888-022-03754-0)
Supplement: Supplementary file 1 — Additional file 1. [file 12888_2022_3754_MOESM1_ESM.pdf]

Appendix A. **Consolidated criteria for reporting qualitative studies (COREQ): 32- item checklist**

Developed from: Tong A, Sainsbury P, Craig J. Consolidated criteria for reporting qualitative research (COREQ): a 32-item checklist for interviews and focus groups. International Journal for Quality in Health Care. 2007. Volume 19, Number 6: pp. 349 – 357

No.	Item	Guide questions/description	Reported on Page no.
**DOMAIN 1: RESEARCH TEAM AND REFLEXIVITY**			
Personal characteristics			
1	Interviewer/facilitator	Which author/s conducted the interview or focus group?	Methods – data collection
2	Credentials	What were the researcher's credentials? E.g. PhD, MD	Methods – data collection
3	Occupation	What was their occupation at the time of the study?	Methods – data collection
4	Gender	Was the researcher male or female?	Methods – data collection
5	Experience and training	What experience or training did the researcher have?	Methods – data collection
Relationship with participants			
6	Relationship established	Was a relationship established prior to study commencement?	Methods – participants and recruitment procedures
7	Participant knowledge of the interviewer	What did the participants know about the researcher? e.g. personal goals, reasons for doing the research	NA
8	Interviewer characteristics	What characteristics were reported about the inter viewer/facilitator? e.g. Bias, assumptions, reasons and interests in the research topic	NA
**DOMAIN 2: STUDY DESIGN**			
Theoretical framework			
9	Methodological orientation and Theory	What methodological orientation was stated to underpin the study? e.g. grounded theory, discourse analysis, ethnography, phenomenology, content analysis	Methods – study design; data analysis
Participant selection			
10	Sampling	How were participants selected? e.g. purposive, convenience, consecutive, snowball	Methods – participants and recruitment procedure
11	Method of approach	How were participants approached? e.g. face-to-face, telephone, mail, email	Methods – participants and recruitment procedure
12	Sample size	How many participants were in the study?	Methods – participants and recruitment procedure
13	Non-participation	How many people refused to participate or dropped out? Reasons?	Methods – participants and recruitment procedure
Setting			

14	Setting of data collection	Where was the data collected? e.g. home, clinic, workplace	Methods – data collection
15	Presence of non-participants	Was anyone else present besides the participants and researchers?	Methods – data collection
16	Description of sample	What are the important characteristics of the sample? e.g. demographic data, date	Results
Data collection			
17	Interview guide	Were questions, prompts, guides provided by the authors? Was it pilot tested?	Methods – data collection
18	Repeat interviews	Were repeat inter views carried out? If yes, how many?	NA
19	Audio/visual recording	Did the research use audio or visual recording to collect the data?	Methods – data collection
20	Field notes	Were field notes made during and/or after the interview or focus group?	NA
21	Duration	What was the duration of the inter views or focus group?	Methods – data collection
22	Data saturation	Was data saturation discussed?	Methods – data collection; data analysis
23	Transcripts returned	Were transcripts returned to participants for comment and/or correction?	NA
DOMAIN 3: ANALYSIS AND FINDINGS			
Data analysis			
24	Number of data coders	How many data coders coded the data?	Methods – data analysis
25	Description of the coding tree	Did authors provide a description of the coding tree?	NA
26	Derivation of themes	Were themes identified in advance or derived from the data?	Methods – data analysis
27	Software	What software, if applicable, was used to manage the data?	Methods – data analysis
28	Participant checking	Did participants provide feedback on the findings?	NA
Reporting			
29	Quotations presented	Were participant quotations presented to illustrate the themes/findings? Was each quotation identified? e.g. participant number	Results
30	Data and findings consistent	Was there consistency between the data presented and the findings?	Results
31	Clarity of major themes	Were major themes clearly presented in the findings?	Results
32	Clarity of minor themes	Is there a description of diverse cases or discussion of minor themes?	Results
