# Supplementary material for: Perceived mental illness stigma among family and friends of young people with depression and its role in help-seeking: a qualitative inquiry
Source: BMC Psychiatry. 2022 Feb 11;22:107. doi: 10.1186/s12888-022-03754-0 (PMC8832742; doi:10.1186/s12888-022-03754-0)
Supplement: Supplementary file 2 — Additional file 2. [file 12888_2022_3754_MOESM2_ESM.docx]

Appendix B. Codebook for ‘Perceived stigma towards mental illness and lack of support among family and friends’

1. **Negative beliefs towards depression and individuals who experience it**

| **Detailed Description** | Negative beliefs towards mental illness and individuals with mental illness that are endorsed by family members and friends, as well as their perceived nature, causes, and prognosis of the illness. |
| --- | --- |
| **Inclusion Criteria** | Any mentions of:   1. Perceived negative characteristics of individuals with mental illness (e.g., lazy, weak, dishonest, crazy, violent, abnormal) 2. Perceived causes of mental illness (e.g., supernatural influences, character flaws) 3. Perceived nature and prognosis of mental illness (e.g., temporary, not a serious illness) 4. Perceived control over experience of mental illness |
| **Exclusion Criteria** | NA |
| **Exemplars** | “Yah so to them it’s just uh, like a… like I choose to feel sad for as long as that period lasts it’s my choice and I can always choose not to feel depressed.”  “they don’t understand… oh schizophrenia, oh crazy. Depression, oh cannot *tahan* (handle) the stress or what, like that”  “the lazy disease”  “Their point of view is very skewed *la* in a way. Because they don’t see it as an illness they see it as emotions you see. So that’s why they were like oh you feeling like this you giving up it’s just you and not having faith” |

1. **Negative reactions and behaviours towards individuals with depression**

| **Detailed Description** | Negative reactions and behaviours towards individuals with mental illness by family members and friends |
| --- | --- |
| **Inclusion Criteria** | Any mentions of:   1. Trivialising experience of depressive symptoms 2. Mocking or discriminating individuals with mental illness 3. Other negative attitudes or behaviours towards individuals with mental illness |
| **Exclusion Criteria** | NA |
| **Exemplars** | “Because they either make fun or just be disgusted... It’s hard lah…”  “No because, I don’t know how they know that I have this major depression that I’m seeking treatment at IMH, so when they know, there’s rumours spreading that I’m insane.” |

1. **Lack of appropriate support from family and friends**

| **Detailed Description** | Failure of family members and friends to provide support at an appropriate level of urgency or importance. |
| --- | --- |
| **Inclusion Criteria** | Any mentions of:   1. Moments of hesitation or inaction to seek formal help 2. Dismissing/avoiding/denying the condition 3. Showing lack of understanding and empathy 4. Providing unhelpful support (e.g., ill advice, inappropriate comparisons, blaming individuals) |
| **Exclusion Criteria** | NA |
| **Exemplars** | “Even if I told them they wouldn’t take it seriously. Like after I tried to kill myself I went to them and then they simply brushed it aside, just as a reaction to stress”  “like let’s say I feeling upset or what then they just keep quiet.”  “It’s just that my family has always been very hesitant. Not because the therapists are bad but because they are just having trouble dealing with it. Or coming to terms with what they are having to do. It’s a lot of shame, it’s a lot of denial.”  “my friend said that “uh, that all these problems are just very small” and I should like just stop whining you know that kind of thing.”  “All of depression is about feeling sad. Just don’t feel sad, ah!” |

1. **Stigmatizing beliefs towards seeking treatment**

| **Detailed Description** | A lack of trust of psychiatry as a whole by family members and friends, including the perception that treatments and therapies are a scam, ineffective, and will not help. |
| --- | --- |
| **Inclusion Criteria** | Any mentions of:   1. Distrust towards the practice of psychiatry 2. Distrust towards medications for mental illness 3. Distrust towards healthcare professionals 4. Stigma associated with seeking/receiving treatment for mental illness 5. Stigma associated with being treated at a tertiary hospital 6. Alternative treatment as being more reliable than psychiatric treatment |
| **Exclusion Criteria** | NA |
| **Exemplars** | “my father even say things like ultimately at the end of the day, the doctor need patients to earn money mah so like, yah so you cannot believe all the things that the doctor say one yah because they just want to keep you as their patient to earn money”  “Again they don’t, they, they’re really old school, they don’t agree with medication, they think I’m wasting my money on a therapist, they think that I just need to pray and then everything will be ok.”  “Because, I guess it’s more of they still you know… no matter what it’s still Singapore – Asian country. There’s still stigma to it *lah*. So to get warded in IMH you know like there’s a certain level of stigma to it *lah*, attached to it, yeah.” |

1. **Impact of stigma on individuals**

| **Detailed Description** | Impact of stigmatizing and trivializing attitudes on the individual, on the way they think and their behaviours, and also the way they explain their illness to others. |
| --- | --- |
| **Inclusion Criteria** | Any mentions of:   1. Feelings from experiencing stigma 2. Its influence on their thinking/behaviour such as believing that there is something wrong with themselves etc. 3. Stigma experienced changing the way they think about their illness, the way they tell others about their illness, or the way they manage their illness (e.g., hiding it, brushing it off, or feeling ashamed of illness), |
| **Exclusion Criteria** | NA |
| **Exemplars** | “I think they don’t accept it, I don’t accept it. That’s why I always feel like there’s something wrong with me, I’m damaged goods”  “Because of that I didn’t really tell anybody. I kind of isolated myself and I was just afraid to share with people.”  “Even though I did try to seek help with my…family…they…they say uh…somewhere along the lines of just get over it or something like that. Pretty annoying, so then I just don’t talk about it anymore lah with them.” |
